# Case Report: Membranous Nephropathy Secondary to Cobalamin C Disease

**DOI:** 10.3389/fmed.2021.807017

**Published:** 2022-01-21

**Authors:** Qiang Wang, Qi Wang, Yanxia Gao, Chenquan Tang, Zhaoli Gao, Zhao Hu

**Affiliations:** ^1^Department of Nephrology, Qilu Hospital (Qingdao), Cheeloo College of Medicine, Shandong University, Qingdao, China; ^2^Department of Nephrology, Suzhou Integrated Traditional Chinese and Western Medicine Hospital, Suzhou, China; ^3^Department of Nephrology, Qilu Hospital, Cheeloo College of Medicine, Shandong University, Jinan, China

**Keywords:** secondary membranous nephropathy, cobalamin C disease, MMACHC, methylmalonic academia, hydroxylcobalamin

## Abstract

**Background:**

Mutation of *MMACHC* causes inherited cobalamin C disease with methylmalonic academia (MMA) and homocysteinemia. Renal complications may also be present in patients with this deficiency. However, membranous nephropathy secondary to cobalamin C disease has not been reported to date.

**Case Presentation:**

We encountered a 17-year-old female patient with a trans-compound mutation of *MMACHC* who presented with membranous nephropathy, MMA, homocysteinemia, and hyperuricemia. The mutations of c.80A>G (chr1:45966084) and c.482G>A (chr1:45974520) (predicting p.Gln27Arg and p.Arg161Gln missense changes at the amino acid level) had been inherited from her father and mother, respectively. Hydroxocobalamin, betaine, and L-carnitine were administered. The patient achieved complete remission of the membranous nephropathy and resolution of the MMA, homocysteinemia, and hyperuricemia.

**Conclusion:**

Membranous nephropathy secondary to cobalamin C disease is reversible with timely intervention.

## Introduction

Many glomerular diseases are complicated by poor renal outcomes, especially some inherited diseases such as Alport syndrome. However, certain types of glomerular injury secondary to some inherited diseases may be reversible. Mutation of *MMACHC* causes cobalamin C disease (cblC), which is the most common genetic defect of cobalamin metabolism. The downstream intracellular synthesis of adenosylcobalamin and methylcobalamin, coenzymes for the enzymes methylmalonyl-coenzyme A mutase and methionine synthase, are thus disturbed, causing elevated methylmalonic acid and homocysteine with decreased methionine production. This disorder results in heterogeneous clinical presentations, both early-onset and late-onset, in patients of a wide range of ages. The main features are growth retardation, poor feeding and lethargy, hemolytic uremic syndrome, chronic thrombotic microangiopathy, developmental delay, and progressive encephalopathy and leukoencephalopathy (1).

Renal complications associated with cblC are uncommon and often do not represent the initial presentation, making them more likely to be ignored. Of these complications, thrombotic microangiopathy, chronic tubulointerstitial nephritis, and renal tubular acidosis are commonly reported (2). However, related glomerular diseases are infrequent; only one case of focal segmental glomerulosclerosis and one case of membranoproliferative glomerulonephritis have been reported to date (3, 4). Membranous nephropathy (MN) associated with cblC has not been identified. We herein report a proband who presented with MN secondary to trans-compound mutations of *MMACHC* and was successfully treated with vitamin B replacement therapy.

## Case Presentation

A 17-year-old girl presented to our nephrology department with a 7-month history of intermittent lower extremity edema, proteinuria, and hematuria. An initial renal biopsy performed at another hospital 4 months before presentation to our center indicated possible IgA nephropathy, and she was therefore prescribed monotherapy with the angiotensin receptor blocker valsartan. However, her clinical presentation was refractory to this treatment. She reported no history of drug use, infections, or malignancy and had no family history of hepatitis B or C, HIV, rheumatic disease, or tumors. She was normotensive, and a general physical examination and funduscopic examination were unremarkable. Her lung fields were clear with no rales or fremitus. A 24-h urine protein test revealed a total protein level of 2.75 g. Urinalysis revealed 24 erythrocytes per high-power field. Her serum concentrations of urea, creatinine, and albumin were within normal limits. Autoantibody test results were unremarkable. Serum antiphospholipase A2 receptor antibodies were negative. The serological results are shown in Supplementary Table 1. Renal ultrasound findings were normal.

**Figure 1:**
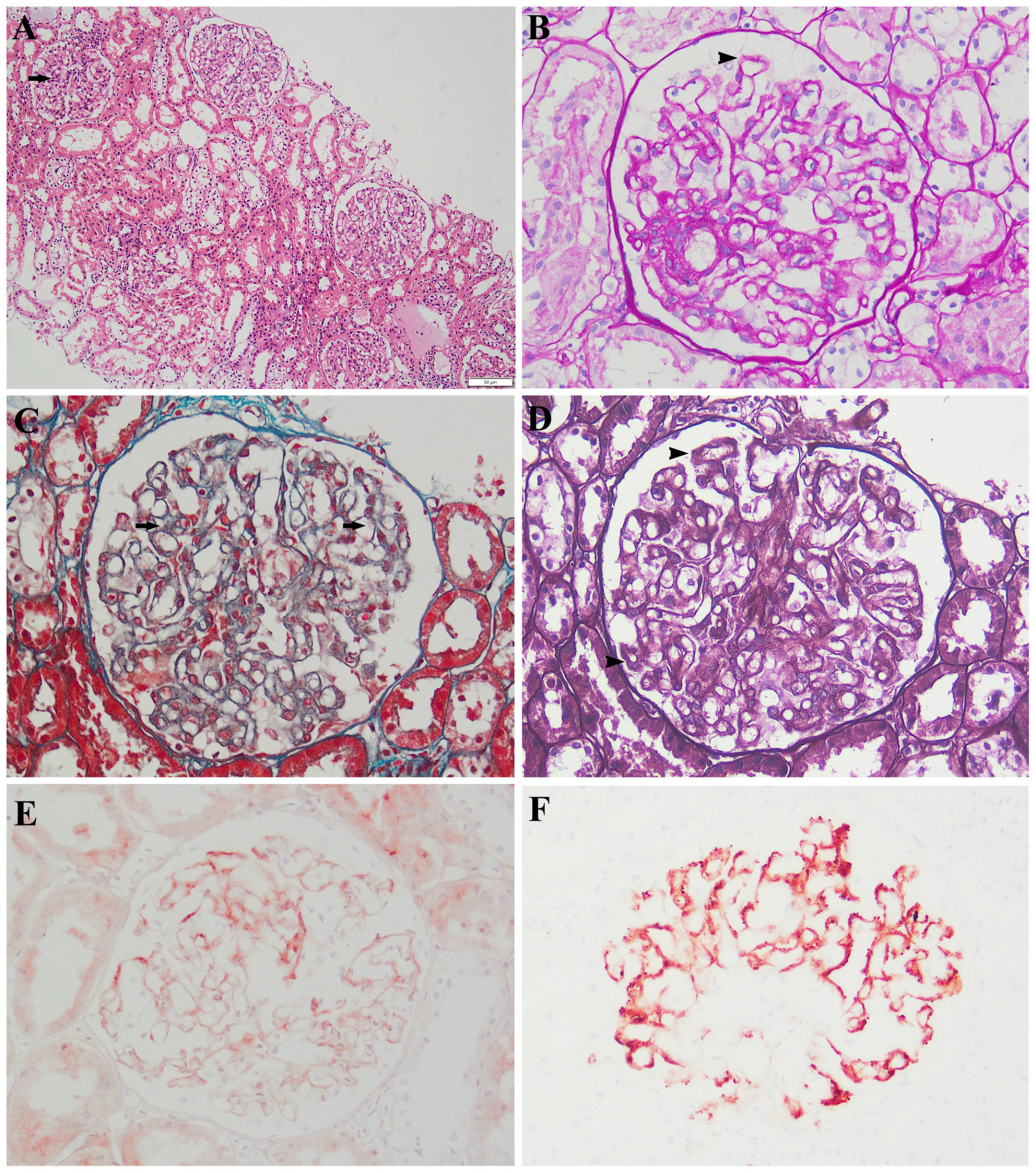
Histologic and immunohistochemical features of renal lesions. **(A)** Segmental mesangial expansion (hematoxylin and eosin; original magnification, ×100). **(B,D)** Slight global glomerular basement membrane thickening (periodic acid–Schiff and Jones methenamine silver, respectively; original magnification, ×200). **(C)** Scattered fuchsinophilic deposits (Masson's trichrome; original magnification, ×200). Immunohistochemical assay (original magnification, ×200) showed **(E)** mild staining for IgG and **(F)** fine granular deposits of c1q.

The histopathological analysis of the previous renal biopsy specimen was revisited. The biopsy revealed 22 glomeruli, 1 (4.5%) of which showed global sclerosis and 1 (4.5%) of which showed focal segmental sclerosis. Mild mesangial expansion, glomerular basement membrane thickening, endothelial swelling, focally swollen podocytes, and hypercellularity were observed (Figure 1). Swelling of the tubular epithelium was also noted. A patchy infiltration of lymphocytes and monocytes was present within the interstitial area. There was no interstitial fibrosis. The capillary walls of the interstitial area showed no lesions. An immunohistochemical assay showed granular deposits along the capillary walls for IgM, C3, C1q, and kappa and lambda light chains as well as mild staining for IgG and IgA. IgG subclass staining showed segmental positivity for IgG1 but negativity for IgG2, IgG3, IgG4, and antiphospholipase A2 receptor.

The granular deposits along the outside of the capillary walls noted on the immunohistochemical assay, especially for lambda and C1q, indicated immune complexes along the capillaries. Spike-like projections were noted at the 3-o'clock position under silver staining, indicating a spiked glomerular basement membrane around the immune complexes. Masson staining showed scattered fuchsinophilic deposits, which denoted immune complexes. The IgG deposits in patients with primary MN are predominantly IgG4, whereas other isotypes have been identified in certain causes of secondary MN (5, 6). In this case, the lesions of mesangial cells, endothelial cells, and podocytes were inconsistent with primary MN. Lupus nephritis was excluded because of the absence of systemic manifestations of lupus, and all immunoserological markers were negative except the antinuclear antibody titer (1:100). Neither a solid tumor nor hematological malignancy was shown by computed tomography or hematological examinations, and all tumor biomarkers were negative. These findings led to the diagnosis of secondary MN.

**Figure 2:**
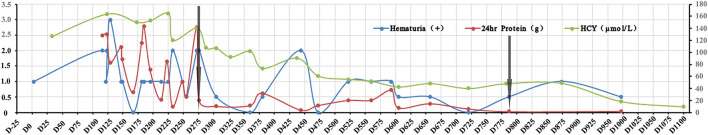
Changes in hematuria, proteinuria, and serum homocysteine during treatment. The X-axis indicates the time points of treatment and follow up, the left Y-axis represents the 24-h urine protein and hematuria, and the right Y-axis represents the serum homocysteine concentration. The green curve denotes the serum homocysteine concentration, the red curve represents hematuria (0.5 indicates + –, 1 indicates +, 2 indicates ++, and 3 indicates + + +), and the blue curve represents the 24-h urine protein. The left arrow indicates the time of initial OHCbl treatment, and the right arrow indicated the time of revised treatment.

**Figure 3:**
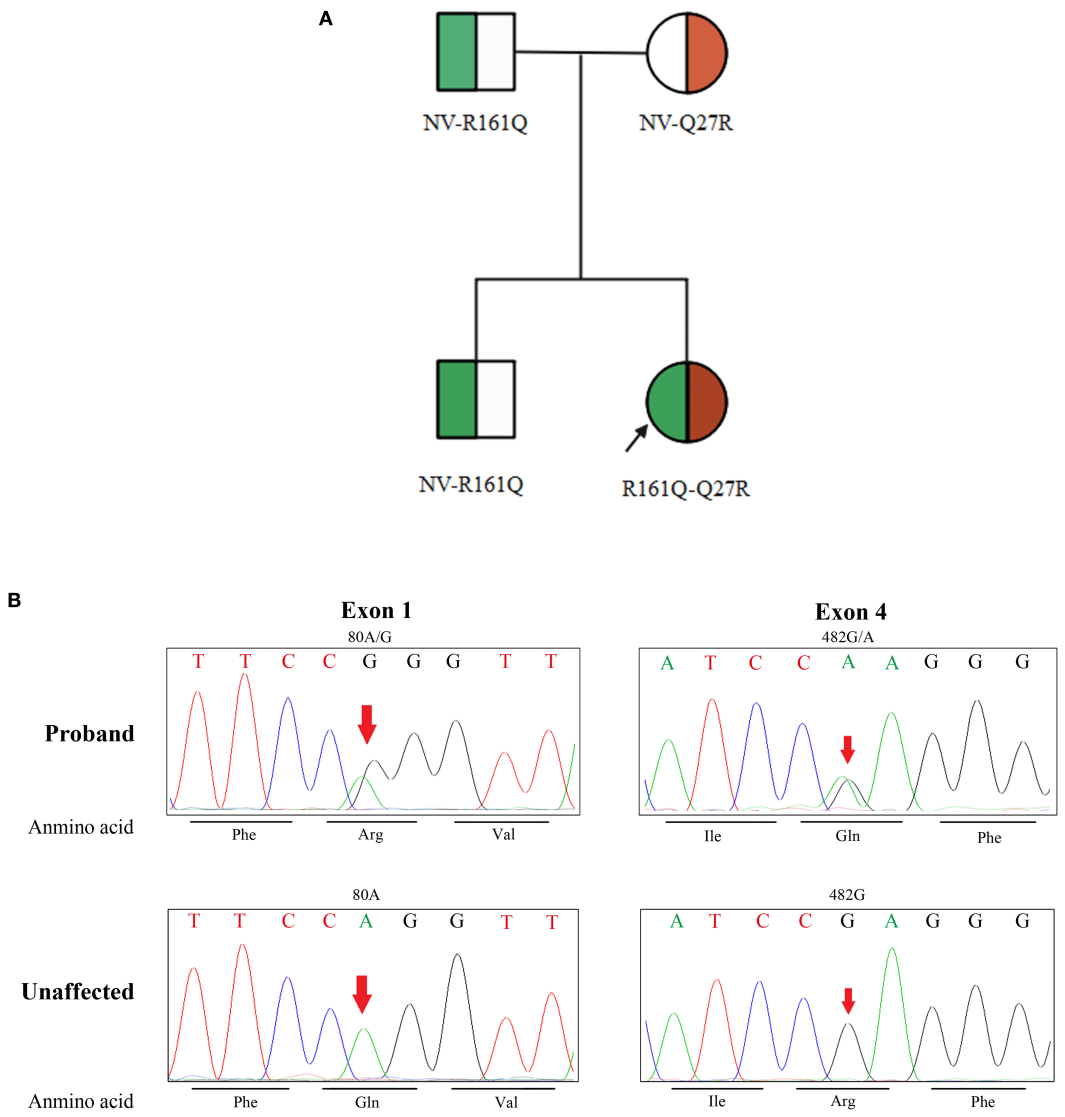
Family pedigree and identification of *MMACHC* mutation. **(A)** Shows the family pedigree. Squares indicate male family members, and circles represent female family members. The arrow indicates the proband. The symbol of both green and red in the circle represent heterozygote. NV, non-variant. **(B)** Shows the mutated locus of the DNA sequences. Phe, phenylalanine; Arg, arginine; Val, valine; Ile, isoleucine; Gln, glutamine; Unaffected, unaffected family members.

The peak serum homocysteine concentration was 164 μmol/L (reference range, <15 μmol/L) (Figure 2), and the uric acid concentration was 625.7 μmol/L (reference range, 50–410 μmol/L). The blood propionyl-L-carnitine concentration was 4.39 μmol/L (reference range, 0.35–3.36 μmol/L), the ratio of propionyl-L-carnitine to acetyl-L-carnitine was 0.38 (reference range, 0.02–0.25), and the urine methylmalonic acid concentration was 5.1 μmol/L (reference range, 0.2–3.6 μmol/L). Genetic testing revealed compound heterozygous mutations of *MMACHC* (*NM_015506*), which confirmed late-onset cblC. The family pedigree is shown in Figure 3A. None of the patient's family members showed clinical symptoms. The proband was a compound heterozygote for two transitions: one in exon 1 (chr1:45966084, c.80A>G) and the other in exon 4 (chr1:45974520, c.482G>A) of *MMACHC* (Figure 3B). These predicted p.Gln27Arg and p.Arg161Gln missense changes at the amino acid level. The proband's father, mother, and brother were heterozygous for Q27R, R161Q, and Q27R, respectively. Therefore, the MN was considered secondary to a cobalamin-related remethylation disorder. The patient was treated with hydroxocobalamin (OHCbl) at 1 mg/week intramuscularly, betaine at 1,000 mg/day, and L-carnitine at 2,000 mg/day orally. Her serum homocysteine concentration gradually decreased, and the proteinuria and hematuria resolved during a period of over 1 year. Because of weight gain, however, the dosages were subsequently changed to OHCbl at 1 mg twice a week intramuscularly, betaine at 1,000 mg twice a day, and L-carnitine at 2,000 mg twice a day orally. At the 36-month follow-up visit, the patient's serum homocysteine concentration was 9.57 μmol/L and her serum uric acid concentration was 330.91 μmol/L (Figure 2). Her urinary protein concentration and erythrocyte count were within the reference ranges. The patient is being followed-up to date.

## Discussion

We have herein described a young female patient with secondary MN associated with compound heterozygous mutations of *MMACHC*. A high serum homocysteine concentration was noted after 4 months of treatment with an angiotensin receptor blocker. Blood and urine organic acid testing as well as genetic sequencing were performed. The pathological features of this case were consistent with secondary MN. The patient had no other relevant history. The findings in this case led to a definitive diagnosis of cblC. However, given the deposition of multiple immunoglobulins and C1q as well as the patient's age, we will continue to follow up this patient because of the possibility of lupus in the future. The patient was treated with OHCbl, betaine, and L-carnitine, effectively resolving her renal lesions, proteinuria, and hematuria. The patient's treatment outcome also supports our conclusion that the glomerular lesions were secondary to a deficiency of cobalamin metabolism.

cblC is an inborn error of cobalamin metabolism with autosomal recessive inheritance. The proband in this case was a trans-compound heterozygote with c.80A>G and c.482G>A mutations from both parents. Neither mutation has been proven to be associated with renal involvement. Neither variant is a single-nucleotide polymorphism, and both are more common in Chinese individuals (7, 8). The function of MMACHC protein is impaired in affected patients, causing methylmalonic academia (MMA) and homocysteinemia. This disorder results in diverse clinical presentations, early-onset or late-onset, in patients of a wide range of ages. Patients with c.80A>G mutation seem to show early-onset signs (8), whereas c.482G>A mutation is usually associated with a late-onset pattern (9). Our patient had a combination of c.80A>G and c.482G>A and developed late-onset signs.

MMA and homocysteinemia are common features of cblC, and the clinical manifestations can be diverse. Neurological symptoms are most often seen. Kidney injury accompanying these initial presentations in patients with early-onset disease would be more likely to be noticed by pediatric doctors (10). Diagnosis of the late-onset type is still challenging. In the present case, the diagnosis took 9 months after initiation of the renal injury. Some patients are not diagnosed until their condition has progressed to irreversible kidney injury (11). We previously described another 20-year-old female patient with IgA nephropathy associated with cblC who presented with neurological symptoms. The patient was homozygous for c.452A>G (p.His151Arg). Her serum creatinine concentration was 3.02 mg/dL by the time of diagnosis (12). It is of great importance to consider these inborn errors of cobalamin metabolism when unexplained renal injury is accompanied by neurological presentations and MMA and/or homocysteinemia.

The treatment of renal complications in patients with late-onset disease is similar to that in patients without renal involvement. The goal is to target the primary disease, normalize the serum methionine and homocysteine concentrations, and resolve the MMA. OHCbl and betaine treatment should be initiated once cblC is suspected (1). Folinic acid and levocarnitine are alternative options in some patients. Treatment by a low-protein diet in patients with cblC is controversial because the homocysteine content in dietary protein is very low (13). Thus, no diet control was applied in the present case. According to the 2021 Kidney Disease: Improving Global Outcomes guideline, complete remission is defined as a first-morning or 24-h protein/creatinine ratio of ≤200 mg/g (≤20 mg/mmol) (or negative or trace dipstick) on three or more consecutive occasions (14). Complete remission of the renal injury and normalization of cobalamin metabolism was achieved in this case. Considering the poor renal outcome of glomerular diseases, it is noteworthy that the glomerular injury secondary to cblC is reversible with timely treatment.

Interestingly, our patient's serum uric acid concentration gradually returned to the reference range as the serum homocysteine concentration decreased. A possible explanation is that purine metabolism is involved in cobalamin metabolism. During the cycle in which methionine is changed to homocysteine, methionine is converted to adenosylhomocysteine (1), and the latter is split into homocysteine and adenosine (15); adenosine is then metabolized into uric acid. When this cycle is blocked by the transformation of homocysteine to methionine, adenosine accumulates, resulting in an increased level of uric acid. However, betaine facilitates the conversion of homocysteine to methionine with ensuing normal function of the cycle. A clinical correlation between homocysteinemia and hyperuricemia has also been established (16). A murine model showed that betaine can reduce the serum uric acid concentration (17). The findings of these previous studies make the above explanation more reasonable.

**Figure 4:**
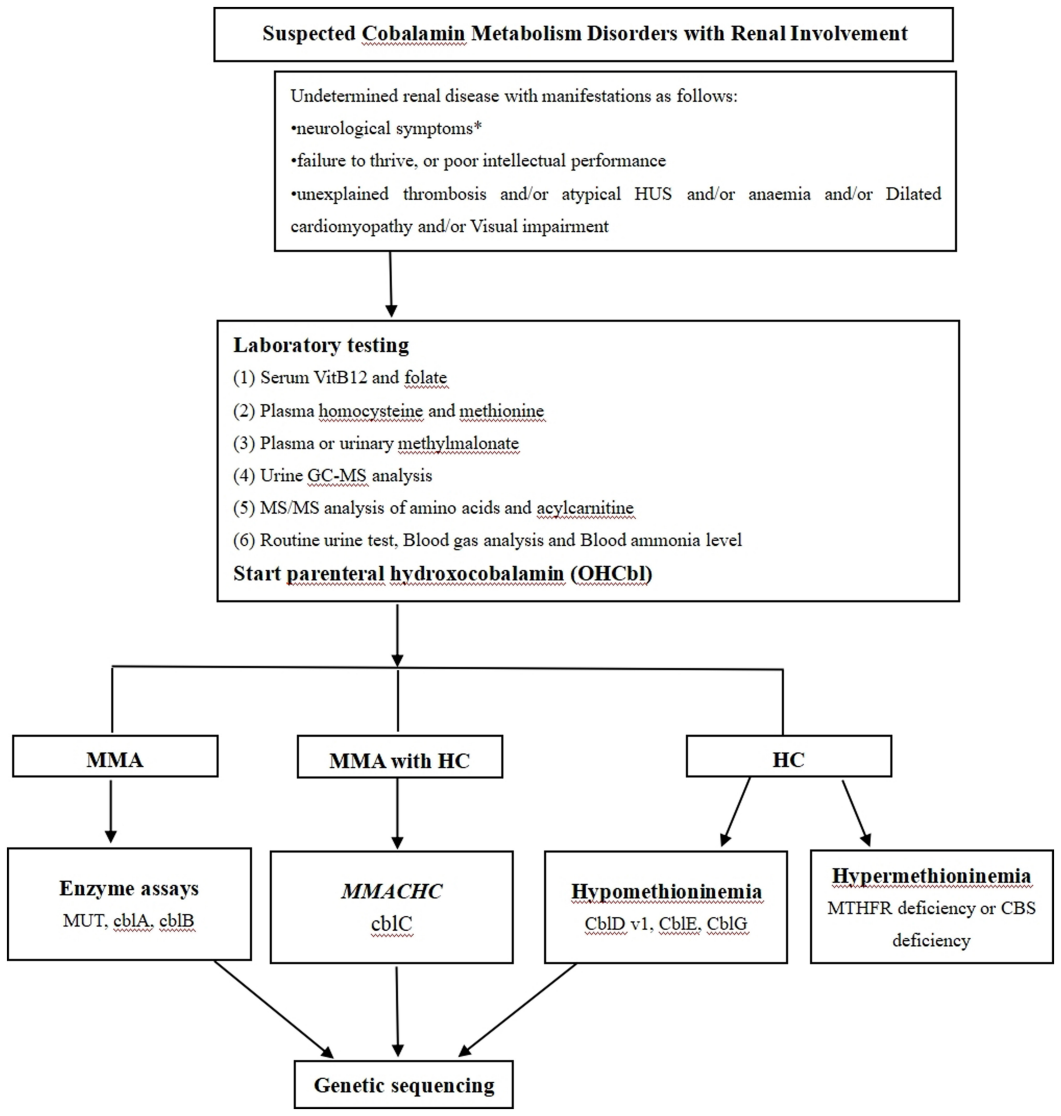
The proposed diagnostic algorithm for suspected cobalamin metabolism disorders with renal involvement. Based on biochemical, enzymatic and genetic complementation analysis, several classes of cobalamin metabolism disorders are known as follows: mut0 /mut- type, cblA type, cblB type, cblC type, cblD-Var1 type, cblD-Var2 type, cblD type, cblF type, cblE type, cblG type, cblJ type, and cblX type; HUS: Hemolytic uremic syndrome; HC: Hyperhomocysteinaemia; MUT: Methylmalonyl-CoA mutase; MMACHC: Gene responsible for methylmalonic acidemia and homocystinuria, cblC type; MTHFR: Methylenetetrahydrofolate reductase; CBS: cystathionine-beta-synthase; * Neurological symptoms include psychiatric symptoms, cognitive impairment, ataxia neuropathy, unsteady gait, myelopathy, as well as seizures.

The high concentration of serum homocysteine was the only clue that led us to suspect cobalamin deficiency in this case. We also propose a diagnostic algorithm for suspected cobalamin deficiency with renal involvement in adult patients (1, 18–20) (Figure 4). This case had two limitations. First, we had no electron microscopy results because no renal tissue was left after performing slicing two times. Second, complete remission was evaluated without renal pathological evidence.

## Conclusion

We have reported the first known case of MN secondary to cblC, which was found to be reversible with timely intervention. Further follow-up and additional cases will be necessary to clarify the pathogenesis of secondary MN.

## Data Availability Statement

The original contributions presented in the study are included in the article/Supplementary Materials, further inquiries can be directed to the corresponding author/s.

## Ethics Statement

The studies involving human participants were reviewed and approved by the Ethics Committee of Qilu Hospital (Qingdao), Cheeloo College of Medicine, Shandong University. Written informed consent to participate in this study was provided by the participants' legal guardian/next of kin.

## Author Contributions

ZH: study design and manuscript revision. QiaW: manuscript writing. YG, QiW, and ZG: data collection. CT: figure editing. All authors contributed to the article and approved the submitted version.

## Funding

This study was supported by the Key Research and Development Project of Shandong Province (2017GSF21116). The funder had no role in the study design, data collection and analysis, decision to publish, or preparation of the manuscript.

## Conflict of Interest

The authors declare that the research was conducted in the absence of any commercial or financial relationships that could be construed as a potential conflict of interest.

## Publisher's Note

All claims expressed in this article are solely those of the authors and do not necessarily represent those of their affiliated organizations, or those of the publisher, the editors and the reviewers. Any product that may be evaluated in this article, or claim that may be made by its manufacturer, is not guaranteed or endorsed by the publisher.

## Abbreviations

cblC: cobalamin C disease
MMA: methylmalonic academia
OHCbl: hydroxocobalamin.
