# Supplementary material for: Case Report: Membranous Nephropathy Secondary to Cobalamin C Disease
Source: Front Med (Lausanne). 2022 Jan 21;8:807017. doi: 10.3389/fmed.2021.807017 (PMC8814342; doi:10.3389/fmed.2021.807017)
Supplement: Supplementary file 1 [file Table_1.DOCX]

Supplementary Table 1. The Laboratory Values of the Patient

| Laboratory Values | Test Results | Reference Range |
| --- | --- | --- |
| WBC(10^9^/L) | 5.83 | 3.5-9.5 |
| HGB(g/L) | 113 | 115-150 |
| PLT(10^9^/L) | 198 | 125-350 |
| CRP(mg/L) | 1.05 | 0-8 |
| ESR(mm/h) | 17 | 0-20 |
| ALB(g/L) | 38.6 | 35.0-55.0 |
| BUN(mmol/L) | 4.4 | 2.30-7.80 |
| Scr(umol/L) | 75 | 44-80 |
| Cystatin C (mg/L) | 0.92 | 0.4-1.1 |
| UA(umol/L) | 519 | 150-350 |
| Glu(mmol/L) | 5.04 | 3.9-6.1 |
| LDH(U/L) | 160 | 91-245 |
| TSH(uIU/ml) | 1.453 | 0.55-4.78 |
| Anti-PLA2R Ab (RU/ml) | 2.7 | ＜20 |
| Anti-GBM Ab (U/ml) | 6.00 | 0-20 |
| Anti-PR3 Ab(U/ml) | 1.20 | 0-5 |
| Anti-MPO Ab(U/ml) | 1.00 | 0-5 |
| Anti-Sm Ab | Negative | Negative |
| ANA | Negative | Negative |
| Anti-ds DNA(IU/ml) | Negative | 0-100 |
| IgG(g/L) | 10.80 | 7.0-16.0 |
| IgA(g/L) | 1.79 | 0.7-4.0 |
| IgM(g/L) | 1.24 | 0.4-2.3 |
| C3(g/L) | 0.83 | 0.9-1.8 |
| C4(g/L) | 0.219 | 0.1-0.4 |
| HBsAg(COI) | 0.435 | 0-1.0 |
| HCVAg (S/CO) | 0.13 | 0-1.0 |
| TPHA (S/CO) | 0.060 | 0-1.0 |
| HIVAb (COI) | Negative | ＜0.9 |

WBC: White Blood Cell; HGB: Hemoglobin; PLT: Platelet ; CRP: C Reactive Protein; ESR: Erythrocyte Sedimentation Rate; ALB: Albumin; BUN: Blood Urea Nitrogen; Scr: Serum Creatinine; UA: Uric Acid; Glu: Glucose; LDH: Lactate Dehydrogenase; TSH: Thyroid Stimulating Hormone; Anti-PLA2R Ab: Anti-Phospholipase A2 Receptor Antibody; Anti-GBM Ab: Anti-Glomerular Basement Membrane Antiboby; Anti-PR3 Ab: Anti-Proteinase 3 Antibody; Anti-MPO Ab: Anti-Myeloperoxidase Antibody; Anti-Sm Ab: Anti-Sm Antibody; ANA: Antinuclear Antibody; Anti-ds DNA: Anti-double-stranded DNA; HBsAg: Hepatitis B Surface Antigen; HCVAg: Hepatitis C Virus Core Antigen; TPHA: Treponema Pallidum Haemagglutination Assay; HIVAb: Human Immunodeficiency Virus Antibody.
